# Urinary Neutrophil Gelatinase Associated Lipocalin (NGAL) for Early Diagnosis of Acute Kidney Injury in Renal Transplant Recipients

**DOI:** 10.5812/numonthly.9385

**Published:** 2013-03-30

**Authors:** Zohreh Rostami, Mohammad Nikpoor, Behzad Einollahi

**Affiliations:** 1 Nephrology and Urology Research Center, Baqiyatallah University of Medical Sciences, Tehran, IR Iran

**Keywords:** Neutrophil Gelatinase-associated Lipocalin Protein, Rat, Acute Kidney Injury, Kidney Transplantation

## Abstract

**Background:**

Early predictive biomarkers for acute kidney injury (AKI) such as neutrophil elatinase-associated lipocalin (NGAL) could identify patients who may benefit from early initiation of treatment.

**Objectives:**

We aimed to obtain a cut off point for AKI prediction by urine NGAL in kidney transplantation.

**Patients and Methods:**

In a prospective cohort study, 64 adult who underwent kidney transplantation from living or deceased donors at Baqiyatallah transplant center between April 2009 and January 2010 were included. Patients divided into two groups based on the presence or absence of graft dysfunction. In this study, early graft dysfunction (post transplantation AKI) was defined as Cr level more than 1.5 mg/dL on the second postoperative day.

**Results:**

Post-transplant AKI was observed in 31 recipients. Mean urinary NGAL level was greater in recipients with AKI (P = 0.024). In linear regression model, AKI was only factor affected on urinary NGAL level (B = 299.8, P = 0.009). The best sensitivity and specificity for AKI detection by urinary NGAL observed at 2 hour after operation with cut-off point 204 ng/mL.

**Conclusions:**

Our study showed in those who developed early post transplantation graft dysfunction the best AUC-ROC for urine NGAL observed at a cut-off value of 204 ng/mL after 2 hour post transplantation.

## 1. Background

Acute kidney injury (AKI) occurs to some extent almost always in deceased kidney transplants due to renal ischemia-reperfusion, and even in some live donor allografts, and commonly causes varying degrees of early renal allograft impairment (1, 2) that predisposes patients to acute and chronic rejection (2). Therefore, early predictive allograft dysfunction biomarkers could identify patients who may benefit from early initiation of treatment (3).

According to the RIFLE criteria, AKI was defined as an abrupt increase in serum Cr of at least to more than 150% (1.5-fold from baseline) and traditionally identified by measurement of serum creatinine (Cr) (4). Unfortunately, Cr is an unreliable indicator for AKI, and it cannot timely and accurately reflect situation of renal function unless a steady state has been obtained, which may require several days (1). Thus, considerable rises in serum Cr does not appear until 48-72 h after initial insult (5). However, several studies (4, 6, 7) have revealed that neutrophil gelatinase-associated lipocalin (NGAL) has significantly increased in urine and plasma among patients with AKI, NGAL increment occurs 24–48 h before the rise in serum Cr (5, 8). NGAL is emerging as a convenient biomarker for predicting AKI even in patients with multiple comorbid disease and with unknown point in time from the beginning of kidney injury (9).

In addition, one of the necessities for clinical use of any AKI biomarkers is a cut-off determination for defining abnormality (7). Recently, few studies (6, 10-12) reported different sensitivity, specificity and AUCs for the diagnosis of AKI, which are essential to determine the accuracy of the biomarkers (8). So, in order to improve and appraise strategies for the prevention and treatment of AKI, there is a great need for further evaluation for estimation of accurate cut-off point for NGAL in kidney transplant recipients. Comparing with previous studies (13-15) which considered plasma NGAL as an early and sensitive biomarker of the AKI commencement, it is appropriate to assess urinary NGAL in renal transplant recipients as a special model. On the other hand, several studies with different age, gender and race should be performed for decision-making cut-off.

## 2. Objectives

In this study, we conducted a prospective study to examine the results of the few earlier studies that evaluated diagnostic value of urinary NGAL in the prediction of early graft dysfunction in kidney transplant recipients among Iranian population. As urinary NGAL has been suggested to represent kidney damage better than plasma NGAL, we chose urine as sample material (16). We aimed to obtain a cut off point for NGAL until identify early graft dysfunction in a healthy kidney which transplanted to the other individual (17).

## 3. Patients and Methods

### 3.1. Participants

In a prospective cohort study, 64 adult patients aged more than 18 years old were consecutively included. They underwent kidney transplantation for the first time at Baqiyatallah transplant center between April 2009 and January 2010. The majority of patients (86%) received a kidney from a living donor. Patients who had history of recent (within one month ago) infectious diseases or inflammatory disorders were excluded from the operation and subsequently from the study. Patients divided into two groups based on the presence or absence of early graft dysfunction. The proposal of this study was approved by the local ethics committee of Baqiyatallah university of medical sciences and written informed consent was given from all patients.

### 3.2. Immunosuppressive Regimen

All patients received triple immunosuppressive therapy: (1) Cyclosporine (targeting a trough level of 200 to 300 ng/mL for first 3 months, 100 to 250 ng/mL for 4 to 12 months and 100 to 150 ng/mL thereafter); (2) Mycophenolate mofetil (1-2 g per day) or Azathioprine (1-2 mg/kg per day) and (3) Prednisolone (initially 1 mg/kg daily with tapering to 5-10 mg per day during 3 to 6 months). Antithymocyte globulin (ATG) was routinely administered in highly sensitized patients.

### 3.3. Clinical Parameters of Allograft Function

Early kidney allograft function was monitored by daily measurement of serum Cr, urinary out-put (amount of diuresis), and estimated GFR (eGFR) levels within the first 10 days after transplantation. The eGFR values were calculated according to the modification of diet in renal diseases (MDRD) formula (eGFR = 186 × (serum Cr)^-1.154 ^× (age)^-0.203^ × [0.742 if female] × [1.210 if black]). We considered an excellent renal allograft function if a serum Cr level less than 1.5 mg/dL on the second postoperative day. According to previous studies (6, 16, 18, 19), kidney function after transplantation divided into three groups including immediate graft function (IGF), slow graft function (SGF), and delayed (DGF) graft function. Acute renal failure due to DGF defined as the need for dialysis within the first week after transplantation (1), while AKI due to SGA defined as recipients who do not have a rapidly falling serum Cr level after transplantation, but do have sufficient kidney function to avoid the need for dialysis (19). Therefore in this study, early graft dysfunction or post transplantation AKI was defined as Cr level more than 1.5 mg/dL on the second postoperative day.

### 3.4. Clinical and Biochemical Data Collection

Data collected from all patients included age and gender of recipient and donors, donor source (living and deceased), renal function, cold and warm ischemic times, Cyclosporine level, underling disease, blood pressure, urinary out-put, sodium, potassium, calcium, magnesium, AST (aspartate aminotransferase), ALT (alanine aminotransferase), and hemoglobin.

### 3.5. The Urinary NGAL Measurement

We measured the urinary NGAL level in only 10 patients before transplantation, while urine sample could not be obtained in 54 cases because of oligo-anuria. The urinary samples for determination of NGAL levels were taken at 2, 6, 12, 24 and 48 hours after surgery. Almost 5 mL urine sample was collected and immediately centrifuged at 3000 rpm, 4^ º^C, for 5 minutes, and the supernatant was stored at -70^ º^C. In the current study, urinary NGAL was measured by means of a commercially available ELAISA test kit (Antibody Shop, Gentofte, Denmark).

### 3.6. Statistical Analysis

Statistical analyses were performed using the SPSS version 17.0 for Windows. All quantitative data have been expressed as mean ± SD and the qualitative variables have been shown by percentage. Repeated measurement and ANOVA was used to evaluate the differences of markers between two groups. Independent variables with a P value ≤ 0.2 in the univariate analysis were entered into the multivariate linear regression model.

To measure the sensitivity and specificity of urinary NGAL at different cut-off values, a conventional receiver operating characteristic (ROC) curve was generated at 2, 6, 12, 24 and 48 hours after kidney transplantation. We calculated the area under the curve (AUC) to ascertain the quality of urinary NGAL as an early graft dysfunction diagnostic biomarker. An area of 0.5 is no better than expected by chance, whereas a value of 1.0 signifies a perfect biomarker. The optimal cutoff level was defined by the largest sum of sensitivity and specificity. P value less than 0.05 was statistically considered significant.

## 4. Results

### 4.1. Demographic and Laboratory Characteristics of Recipients

We enrolled 64 kidney transplant recipients. The most common known causes of end stage renal disease were hypertension (37.5%) and diabetes mellitus (10.9%). Table 1 demonstrates demographic and laboratory characteristics.

**Table 1.** Demographic and Laboratory Characteristics of Recipients

Charactristics	
**Gender **	
Male	46 (71.9)
Female	18 (28.1)
**Primary disease number (%)**	
ADPKD ^a^	3 (4.7)
Diabetes mellitus	7 (10.9)
Hypertension	24 (37.5)
Renal stone	1 (1.6)
Unknown	29 (45.3)
**Urinary out-put (mL/24 h)**	4300 ± 1406
**Age, y**	40.4 ± 14.1
**Serum creatinine (mg/dL)**	
Pre-transplantation	8.5 ± 2.5
2 weeks after transplantation	2.1 ± 0.9
**Hemoglobin (g/dL)**	
Pre-transplantation	11.2 ± 1.7
2 weeks after transplantation	10.6 ± 1.8
**Dialysis vintage, mo**	20.1 ± 19.8
**Cold ischemic time, min**	17.3 ± 1.4
**Warm ischemic time, min**	16.8 ± 1.1
**Cyclosporine dose (mg/d)**	368 ± 58
**Magnesium (mg/dL)**	2.14 ± 0.58
**Cyclosporine through level (ng/mL)**	271 ± 143
**Cyclosporine 2 hour post dose (ng/mL)**	505 ± 288
**Uric acid (mg/dL)**	5.06 ± 1.39
**Aspartate aminotransferase (AST) (units/L)**	34 ± 51
**Alanine aminotransferase (ALT) (units/L)**	37 ± 31
**Phosphorus (mg/dL)**	3.0 ± 0.9
**Calcium (mg/dL)**	8.48 ± 0.54
**Fasting blood sugar (mg/dL)**	117 ± 45
**Potassium (mmol/L)**	4.2 ± 0.3
**Sodium (mmol/L)**	136.5 ± 3.5

^a^Abbreviation: ADPKD, autosomal dominant polycystic kidney disease

### 4.2. Demographic and Laboratory Characteristics of Donors

The mean age of donors was 28.6 ± 6 years with the mean hemoglobin level of 15.4 ± 1.1 g/dL and mean serum Cr concentration of 0.9 ± 0.1 mg/dL. A total of 55 patients received a kidney from a living donor, while 9 transplants were carried out using a kidney from a deceased donor. Male to female ratio was 3.9:1 (79.7% vs. 20.3%).

### 4.3. AKI Group Versus Non- AKI Group

More than 50% of our patients had excellent renal allograft function. Post-transplant AKI was observed in 31 recipients including 18 patients who had induction therapy with ATG, 4 cases with DGF. Twenty-four subjects had serum Cr level of more than 1.5 mg/dL after 48h of transplantation. The patients with AKI had higher donor age (P = 0.03), greater post-operative serum phosphorus value (P = 0.03) and lower serum calcium (P = 0.03) and hemoglobin levels (P = 0.02) compared to no-AKI individuals (Table 2). There were no statistically significant differences in other variables between two groups (Table 2).

**Table 2.** Comparison Between Two Groups

	Without AKI Number (%)	With AKI Number (%)	P value
**Recipient gender**			0.1
Female	12 (66.6)	6 (33.3)	
Male	21 (45.7)	25 (54.3)	
**Donor gender**			0.4
Female	2 (50)	2 (50)	
Male	28 (54.9)	23 (45.1)	
**Hypertensive recipient**			0.4
No	17 (47.2)	19 (52.8)	
Yes	16 (57.1)	12 (42.9)	
**Diabetic recipient**			0.6
No	29 (50.9)	28 (49.1)	
Yes	3 (50)	3 (50)	
**Blood transfusion **			0.5
No	31 (52.5)	28 (47.5)	
Yes	2 (40)	3 (60)	
**Furosemide + manitol infusion during operation**			0.8
No	19 (54.3)	16 (45.7)	
Yes	14 (48.3)	15 (51.7)	
**Donor type**			0.2
Deceased	3 (33.3)	6 (66.6)	
Living	30 (54.5)	25 (45.5)	
**Donor hemoglobin (g/dL)**	15.5 ± 1.2	15.4 ± 1.1	0.6
**Donor creatinine (mg/dL)**	0.92 ± 0.15	0.95 ± 0.14	0.4
**Donor fasting blood sugar (mg/dL)**	89 ± 7	92 ± 10	0.3
**Donor urine out-put (mL/d) **	1544 ± 603	1702 ± 743	0.4
**Donor age, y**	27.0 ± 4.5	30.5 ± 7.1	0.03
**Recipient hemoglobin pre-operation (g/dL)**	11.4 ± 1.5	11.0 ± 1.9	0.4
**Recipient creatinine pre-operation (mg/dL)**	8.18 ± 2.87	8.87 ± 2.12	0.2
**Recipient urine out-put (mL/d)**	422 ± 390	393 ± 357	0.7
**Recipient age, y**	41.7 ± 15.3	39.1 ± 12.9	0.4
**Dialysis vintage, mo**	19.1 ± 20.6	21.2 ± 19.4	0.7
**Cold ischemic time, min**	17.4 ± 1.2	17.2 ± 1.6	0.6
**Warm ischemic time, min**	16.8 ± 1.0	16.8 ± 1.2	0.9
**Sandimon dose (mg/d)**	358 ± 63	379 ± 51	0.1
**Magnesium (mg/dL)**	2.3 ± 0.7	2.0 ± 0.4	0.1
**Cyclosporine 2 hour post dose (ng/mL)**	554 ± 233	444 ± 373	0.6
**Cyclosporine through level (ng/mL)**	306 ± 141	236 ± 139	0.08
**Uric acid (mg/dL)**	4.9 ± 1.5	5.3 ± 1.2	0.3
**Aspartate aminotransferase (AST) (units/L)**	39 ± 70	29 ± 18	0.4
**Alanine aminotransferase (ALT) (units/L)**	33 ± 22	40 ± 38	0.3
**Phosphorus (mg/dL)**	2.7 ± 0.7	3.2 ± 1.1	0.03
**Calcium (mg/dL)**	8.6 ± 0.5	8.3 ± 0.5	0.03
**Fasting blood sugar (mg/dL)**	114 ± 45	120 ± 45	0.5
**Potassium (mmol/L)**	4.2 ± 0.3	4.2 ± 0.3	0.8
**Sodium (mmol/L)**	137.1 ± 3.7	135.9 ± 3.3	0.2
**Creatinine (mg/dL)**	1.57 ± 0.44	2.64 ± 1.06	0.000
**Hemoglobin post-operation (g/dL)**	11.1 ± 1.7	10.1 ± 1.8	0.02
**Systolic blood pressure (mmHg)**	132 ± 12	138 ± 12	0.1
**Diastolic blood pressure (mmHg)**	79 ± 4	110 ± 157	0.2
**Urine out-put (mL/d)**	4277 ± 1289	4323 ± 1541	0.8
**GFR (mL/min)**	55.9 ± 16.2	38.3 ± 17.6	0.01

**Figure 1:**
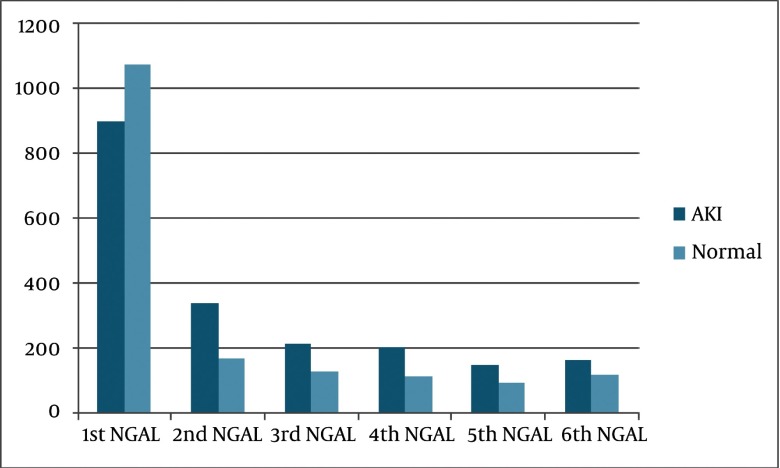
Urinary NGALComparison Between 2 Groups

The mean urinary NGAL level was greater in recipients with AKI as compared to patients who had no AKI (P = 0.024) (Figure 1). In linear regression model, AKI was the only factor affected on urinary NGAL level (B = 299.8, P = 0.009).

The best sensitivity and specificity for AKI detection by serum Cr observed at seven^th^ day Table 3, while by urinary NGAL observed at 2 hour after operation with cut-off point 204 ng/mL (Table 4).

**Table 3.** Sensitivity and Specificity for AKI During Different Day After Kidney Transplantation

	Cut Off Point	Sensitivity %	Specificity %
**Cr 1th day**	1.55	81	37
**Cr 2th day**	1.55	68	81
**Cr 3th day**	1.55	68	81
**Cr 4th day**	1.60	68	78
**Cr 5th day**	1.55	75	81
**Cr 6th day**	1.55	81	84
**Cr 7th day**	1.55	87	87
**Cr 8th day**	1.55	81	75

**Table 4.** Sensitivity, Specificity and Cut off Point for Urinary NGAL Level During Different Hour Post Operation, for AKI Detection

	Cut off Point	Sensitivity, %	Specificity, %	AUC	Confidence Interval	P value
**NGAL ^a^ 2th h**	204	72	67	0.713	0.563-0.863	0.009
**NGAL 6th h**	80	61	40	0.612	0.447-0.778	0.08
**NGAL 12th h**	68	83	43	0.630	0.480-0.780	0.07
**NGAL 24th h**	77	72	64	0.681	0.542-0.820	0.02
**NGAL 48th h**	77	72	63	0.638	0.490-0.785	0.09

^a^Abbreviation: NGAL, neutrophil gelatinase associated lipocalin

Table 3 lists the derived sensitivities, specificities, and predictive values at different cut-off concentrations. The area under the curve (AUC) for 2 hour post-operation urinary NGAL was 0.71 (confidence interval 95% (CI95%): 0.563–0.863; P = 0.009). At the optimal cutoff level of 204 ng/mL, the sensitivity was 72% and the specificity was 67%. AUC-ROC analysis and cutoff level for the best sensitivity and specificity in different hours after transplantation are shown in Table 3.

## 5. Discussion

According to our study, measurement of urinary NGAL was a better predictor for AKI after kidney transplantation than serum creatinine. It is important to note that serum creatinine is not a reliable marker of AKI in kidney transplant patients (1). Therefore, the lack of early biomarkers for graft dysfunction after transplantation may lead to an unacceptable delay in initiating well-timed treatment (1). On the other hand, urine NGAL is significantly higher in patients who develop AKI compared to individuals who do not experience AKI in the early period after transplantation (3, 20). Although a number of studies in which patients experienced AKI have demonstrated the use of both urine and plasma NGAL are powerful independent predictors of AKI with a different proposed cut-offs for optimum utility of this test ranging from 10 to 550 ng/mL (4, 7, 9, 21), there is no clear guidance to offer the using of NGAL in AKI. This wide range could be due to contributing variables such as age, sex, race and preexisting kidney disease which can cause difference to baseline measurement (7).

Our study showed in those who subsequently developed AKI the best AUC-ROC observed at a cut-off value of 204 ng/mL after 2 hour post transplantation, this is in agreement with results obtained by Devarjan et al. (8) and Haase et al. (21) who revealed urine NGAL within 2–6 h after cardiac surgery may predict AKI with a AUC-ROC more than 0.9 and 0.78 respectively. In addition, we found that a cut-off value of 204 ng/mL for urine NGAL at 2 h after operation had the best sensitivity and specificity, while the best sensitivity and specificity for serum Cr level can be observed at least 5-7 day delay in AKI detection.

### 5.1. The Best Timing for Urine NGAL Measurement

**Figure 2:**
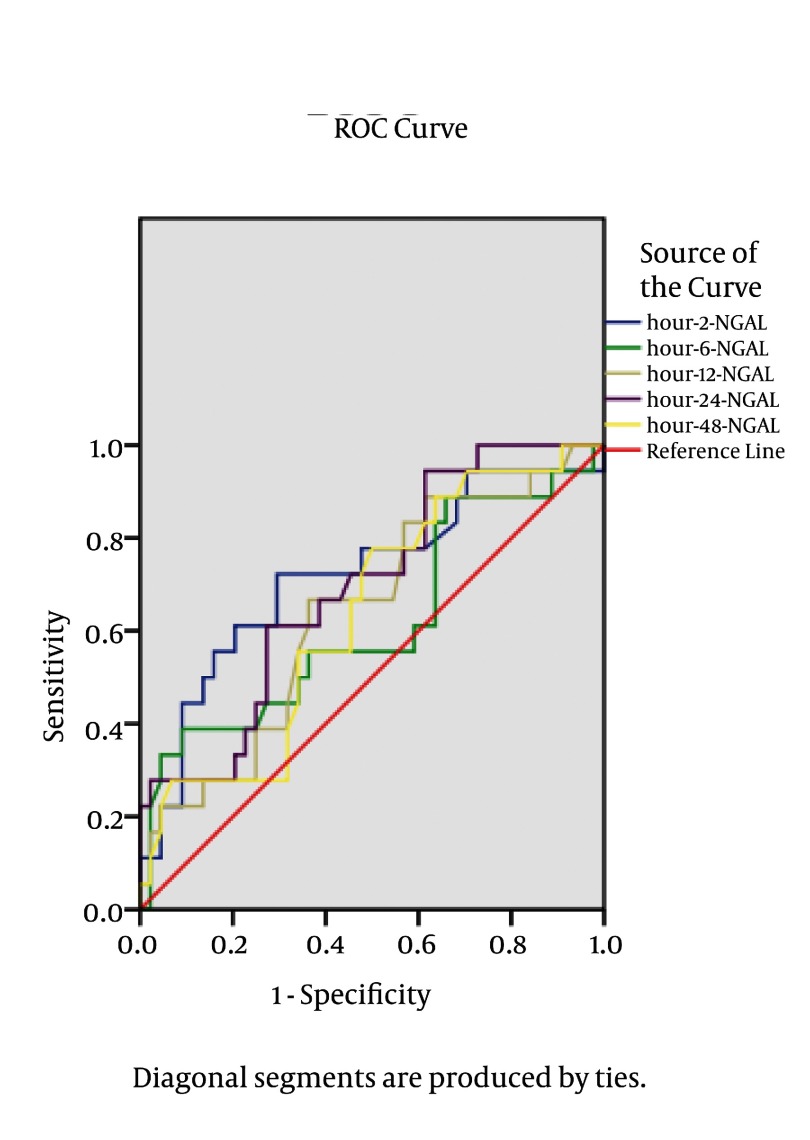
Sensitivity and Specificity of NGAL Based on the Time of Urinary NGAL Measurement

In terms of diagnostic accuracy, Dent et al. (22) demonstrated that the best AUC, sensitivity and specificity of plasma NGAL measurement for prediction of AKI (0.96, 0.84 and 0.94, respectively) were achieved at 2 h after cardiac surgery in a cut-off value of 150 ng/mL. In children undergoing cardiac surgery, the severity of AKI was also correlated with the increase in urine NGAL levels detected at various time points after cardiopulmonary bypass. The urine NGAL at 2 h post-operation had an AUC of 0.95, sensitivity of 0.79, and specificity of 0.92 for prediction of AKI using a cut-off value of 150 mg/mL (8, 23). These findings have been confirmed in prospective studies of adults who experienced AKI following cardiac surgery, in whom urinary NGAL was considerably increased by 1 to 3 hours after the operation (17, 21). In a prospective multicenter study of adults and children undergoing kidney transplantation, urine and plasma NGAL levels in samples collected on the day of transplantation identified those who subsequently developed delayed graft function (which typically occurred 2 to 4 days later), with an AUC of 0.9 for urine NGAL (17). In the intensive care setting, urine and plasma NGAL measurements predict AKI about 2 days prior to the rise in serum Cr, with high sensitivity and an AUC of 0.68 to 0.78 (17, 24). Furthermore, Cullen et al. (7) and Bennett et al. (23) reported patients who subsequently experienced AKI revealed significantly higher urinary NGAL levels immediately during third hour after surgery (Figure 2).

These findings emphasize that the first hour after transplantation could be unique time for AKI revealing by urine NGAL measurement. Thus, NGAL is as an emerging tool could predict development of AKI even in various groups of recipients with multiple comorbid conditions and with unknown timing of initial kidney injury.

### 5.2. NGAL Fluctuation During Early Period of AKI 

According to Figure 1, in excellent allograft function, urinary NGAL value was shown to decrease quickly during first 24 hour post-transplant period and after that slightly incrimination occurred during second day of transplantation. The urinary NGAL levels have previously been shown to be elevated in patients with chronic kidney disease (16). After successful transplantation, however, urinary NGAL must be decreased (25). In the immediate postoperative period, urine output may be important inducing NGAL dilution (13). On the other hand, a kidney transplant recipient in the first hour after operation often has a significant urine output, following aggressive fluid resuscitation, and thus baseline post-transplant NGAL results may be underestimated as a result of urinary dilution (7). So it can explain why urinary NGAL increased in 48^th^ hour.

Furthermore, in contrast to previous studies showed NGAL concentrations vary with age and gender (7), in our study no correlation was detected between urinary NGAL and age and gender.

Limitation: In fact, urinary NGAL might be very dilute early post-transplantation due to high urine out-put (13). Resulting to the synthesis and secretion of NGAL by damaged distal tubule cells, it seems the major fraction of urinary NGAL comes from this segment (8), also damaged tubular epithelium cannot reabsorb urine NGAL, filtered NGAL (8). Subsequently, the high urinary NGAL levels after kidney transplantation might just result from decreased GFR, but also might reflect ongoing damage in the kidney (25) and low urinary NGAL levels in oliguric patients after kidney transplantation suggest that oliguria might also be caused by other reasons, such as suboptimal fluid balance while kidney is in healthy condition (16).

In this study, we concluded urine NGAL at 2 h post-operation had the best sensitivity and specificity for prediction of AKI using a cut-off value of 204 ng/mL. Taken together with the fact, it is clear that the role of NGAL in the diagnosis of post-operative AKI requires further investigation.
